# Central Serous Chorioretinopathy: Treatment with Laser

**DOI:** 10.3390/ph13110359

**Published:** 2020-11-02

**Authors:** Maurizio Battaglia Parodi, Alessandro Arrigo, Pierluigi Iacono, Bruno Falcomatà, Francesco Bandello

**Affiliations:** 1 Department of Ophthalmology, Vita-Salute San Raffaele University, 20132 Milan, Italy; maubp@yahoo.it (M.B.P.); alessandro.arrigo@hotmail.com (A.A.); bandello.francesco@hsr.it (F.B.); 2 TSRetina, 34123 Trieste, Italy; 3 IRCCS-Fondazione Bietti, Via Livenza 3, 00198 Rome, Italy; 4 Department of Ophthalmology, Ospedale Bianchi-Malacrino-Gabrielli, 89100 Reggio Calabria, Italy; bruno.falcomata@alice.it

**Keywords:** central serous chorioretinopathy, laser treatment, subthreshold laser

## Abstract

Currently, no general consensus exists regarding the management of central serous chorioretinopathy (CSC). Laser treatments include three different therapeutic approaches: conventional laser, subthreshold laser and photodynamic therapy. Conventional focal laser, addressed to seal the leaking points, as evidenced on fluorescein angiography, was largely used in the past, but now, it is almost completely abandoned, owing to the potential complications. Several studies confirmed the positive effects achieved by subthreshold laser treatment in CSC, even though its improper application in the PLACE trial has questioned the effectiveness.

## 1. Introduction

**Figure 1:**
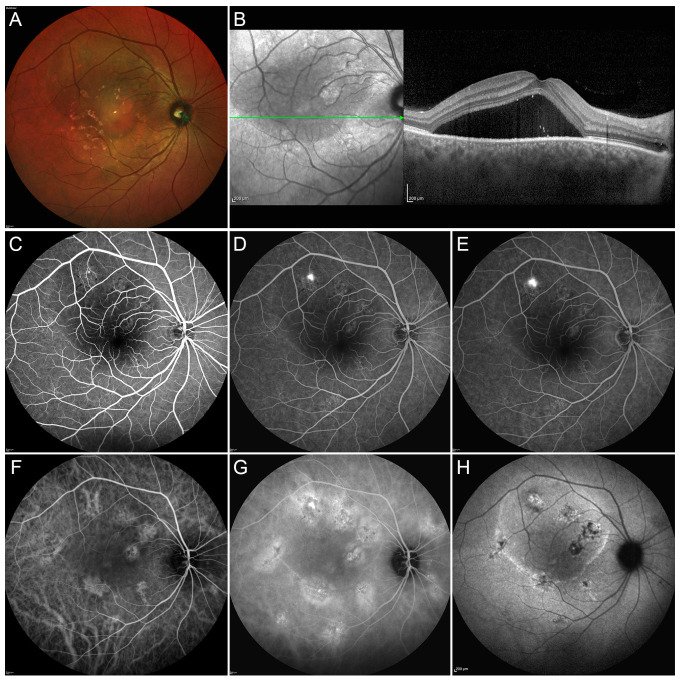
Multimodal Imaging in a case of acute central serous chorioretinopathy (CSC). Multicolor image (**A**) shows the presence of a massive subretinal fluid accumulation involving a big part of the posterior pole. Structural optical coherence tomography OCT scan (green arrow) (**B**) confirms the presence of a large subretinal exudation associated with a pachychoroid. Fluorescein angiography (FA) discloses a region of hypofluorescence, secondary to the masking effect caused by the subretinal fluid, with a well-defined leaking point and no other detectable alterations in the early, intermediate and late phases (**C**–**E**, respectively). The early phase of indocyanine green angiography ICGA (**F**) discloses the presence of multiple alterations of the choroidal vascular network, better highlighted in the intermediate phase of the examination (**G**). Fundus autofluorescence demonstrates the hyperfluorescence corresponding to the active leaking point and the combination of hypo/hyper-fundus autofluorescence in the site of retinal pigment epithelium decompensation (**H**). This case was simply monitored until complete spontaneous resolution of the neurosensory detachment.

Central serous chorioretinopathy (CSC) is a complex disease whose pathogenesis is not fully understood. It is characterized by a serous neurosensory retinal detachment (NSD) that mainly involves the macular area, and it is secondary to the accumulation of subretinal fluid. A dysfunction in the activity of the retinal pigment epithelium (RPE) and an increased vascular choroidal hyperpermeability seem to play a decisive role in the fluid collection (Figure 1).

The acute forms are usually self-limited, with spontaneous resorption of the subretinal fluid in most patients within three months; however, the chronic persistence of the fluid leads to atrophy of the photoreceptors and of the RPE, potentially resulting in a severe central visual loss. Many factors contribute to the manifestation of this common retinal disorder. Personality alterations in association with CSC have been repeatedly demonstrated. An imbalance of sympathetic-parasympathetic activity has also been reported with a possible altered modulation of the choroidal blood flow regulated by the autonomic nervous system. The role of corticosteroids is especially important; whatever the route of administration—inhaled, intranasal, epidural, intra-articular, transcutaneous or periocular—steroids have been associated with an increase in occurrence, persistence, recurrence and worsening of clinical manifestations associated with CSC. Finally, hypertension and cardiovascular disorders are also reported in greater frequency in association with CSC, suggesting that vascular dysfunctions of a more general nature are probably responsible for alterations in the choroidal bed flow described in the CSC.

Although there is no general consensus regarding the most appropriate treatment algorithm, several treatment approaches have been proposed, including observation, diuretics, photodynamic therapy and laser application [1]. 

The aims of an ideal treatment should take into account the resolution of the subretinal fluid, the correction of the vascular permeability alterations and the restoration of the photoreceptor and retinal pigment epithelium (RPE) cells. Whereas photodynamic therapy (PDT) may have a direct impact on the choroidal vascular permeability, leading to a faster resolution of the subretinal fluid, a laser approach can show an effect just on the RPE. In particular, it would be precisely the thermal stimulation of the RPE to lead to a restoration of the blood-retinal barrier, to a regulation of growth factors, including permeability factors, and to the activation of heat shock proteins (HSP).

Overall, the laser treatment of CSC can be subdivided into two categories: conventional or damaging laser treatment, subthreshold/subvisible, or nondamaging laser treatment.

## 2. Results

### 2.1. Conventional Laser Treatment

Focal laser photocoagulation of the leaking points, as identified on fluorescein angiography, has been widely used in the past decades, in the practical management especially of acute CSC. The treatment determines a “sealing” of the RPE defects with potential stimulation of the RPE pump function. Experimental models showed that adjacent RPE cells tend to spread out to fill the defects after laser application, renovating the RPE barrier [2,3]. Treatment is performed applying one to three low-to-moderate intensity burns to the leakage site in an attempt to produce a mild whitening. 

Focal laser photocoagulation can effectively promote the resolution of the fluid but, unfortunately, has no clear effects on the visual acuity improvement and on the recurrence rate over the follow-up [4,5,6,7,8]. Conventional laser application for CSC can bring about some complications, including choroidal neovascularization development (less than 10% of the treated cases) [4], atrophic changes with paracentral scotoma and enlargement of the laser scar over the follow-up. Especially these last secondary effects allow to apply the conventional laser treatment only to the extramacular and extrafoveal leaking points with a safety distance of at least 500 microns from the fovea. With the advent of subthreshold laser treatment and photodynamic therapy, conventional laser application has been used less and less in common clinical practice.

### 2.2. Subthreshold Laser Treatment (STLT)

#### 2.2.1. Mechanism of Action

**Figure 2:**
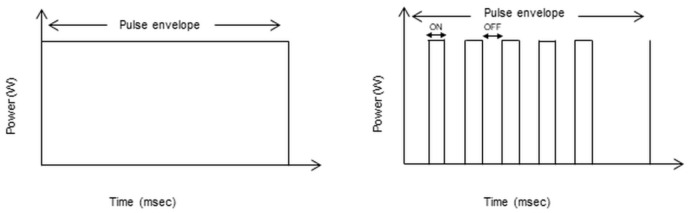
The schematic figure represents the models of laser energy delivery by conventional laser treatment (**left** diagram) and the micropulse mode/subthreshold laser treatment (STLT). In the conventional continuous laser treatment, the energy is delivered in a single pulse, leading to a greater temperature rise in the surrounding retinal layers with subsequent retinal photocoagulation. In the STLT, the laser is delivered in microsecond pulses (**right** diagram). The “on” time is the duration of each micropulse, and the “off” time is the interval between the successive micropulses. The “off” time allows for heat dissipation with the selective activation of retinal pigment epithelium (RPE).

The basic concept underlying STLT is represented by the reduction of the retinal damage due to laser energy, which can be accomplished by decreasing the laser exposure duration and using nonvisible clinical endpoints. In more detail, the laser emission is divided into a “train” of short and repetitive pulses that persist from 0.1 to 0.5 s. The “on” time is the duration of each micropulse, and the “off” time is the interval between the successive micropulses. The “off” time allows for heat dissipation, which ultimately reduces the side effects of conventional laser photocoagulation and limits treatment to RPE. In the laser setting, an activation time (duty cycle) is generally set from 5 to 15 percent. One of the main objectives of the treatment is, therefore, to avoid direct thermal damage to the RPE and the internal retina, characterized in conventional laser photocoagulation by an immediate and visible whitening of the retina due to thermal damage, which can be complicated with the appearance of side effects such as visual field defects, epiretinal fibrosis and choroidal neovascularization in the area of the laser scar (Figure 2). 

**Figure 3:**
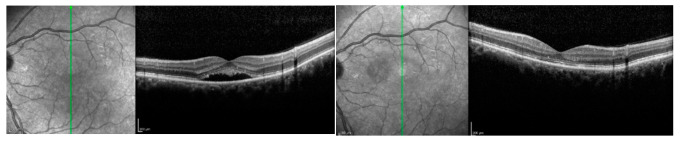
Patient affected by chronic central serous chorioretinopathy treated with subthreshold laser treatment. (**Left**) Optical coherence tomography scan thorough the fovea (green arrow) disclosing a subretinal fluid with photoreceptors elongation before the treatment (visual acuity 20/63). (**Right**) Optical coherence tomography scan of the patient after subthreshold laser application, revealing the complete resolution of the subretinal fluid, with no visible laser scar (visual acuity 20/20).

In particular, practical experience has indicated that, when a short pulse duration is released on the retina, just the RPE is affected, with no inner retinal damage [9]. STLT has been indicated as effective in the management of both acute and chronic CSC by several authors, showing variable rates of subretinal fluid resolution (on average, two-thirds of treated cases) (Figure 3) associated with visual acuity, retinal sensitivity and contrast sensitivity improvements [10,11,12,13,14,15,16,17]. 

However, the comparison of different investigations is hard because of the heterogeneous inclusion criteria, laser wavelength (810 nm and yellow 577 nm) and general setting, along with the extension of the macular area exposed [1].

The STLT, otherwise called nondamaging laser or sublethal laser, would selectively act on the activity of the retinal pigment epithelium by promoting the migration and proliferation of new cells, by activating the release mechanism of the HSP and by promoting the pumping function of RPE cells. Upon the resumption of RPE activity and following the activation of HSP, a progressive but slow reabsorption of the subretinal fluid would occur. Additional advantages of STLT would include the ability to apply the laser near or over the fovea, as well as the option to treat the same area in multiple sessions.

#### 2.2.2. Outcomes and Safety of STLT

To evaluate the effectiveness of STLT in CSC, we operated a literature screening to find clinical studies with at least 12 months of follow-up. Special focus was directed to studies reporting data on visual acuity, retinal sensitivity (RS), percentage of resolution of NSD, number of recurrences, beneficial effects of retreatment and safety of treatment. A summary of the data collection is summarized in Table 1.

Among 68 selected manuscripts dealing with CSC and STLT, five were taken into account for the selected parameters. Two studies had a prospective design.

The mean/median follow-up varied between 12 months and 3.7 years. Overall, the best-corrected visual acuity (BCVA) improved significantly in all studies, with a mean final improvement of 0.1–0.4 LogMar. In parallel, anatomical improvement was registered in 67–100% of patients, with a large proportion of eyes with a complete NSD resolution already present at one month after treatment. Recurrences were noted in at least four studies; in all cases, additional sessions of STLT were administered, leading to a resolution on the NSD recurrences in most cases. As per protocol, in the case of recurrences or NSD persistence, additional STLT was performed, and this resulted in a final mean number of treatments varying between 1 and 2.5. Regardless of the type of laser, 577 or 810 nm, no retinal damage or complications, including choroidal neovascularization (CNV) or laser scar, were observed in association with the laser treatments in the follow-up period.

The results are extremely positive, characterized by a meaningful improvement of visual acuity and a large proportion of anatomical success with NSD resolution. However, the analysis of the treatment protocols shows a great variability in the treatment procedure, so that it is apparently difficult to safely delineate the most appropriate settings.

Lanzetta and Lutrull used STLT with an 810-nm laser. In detail, Lanzetta settled the laser in the following way: spot size of 200 μm, 0.2-s exposure time, duty cycle of 15%, 1.35-Watt power and distribution of treatment with multiple overlapping spots over and adjacent to the area of RPE leak or decompensation detected on fluorescein angiography [11]. With some disparity, Lutrull applied the laser with contiguous spot applications of 200-μm spot sizes, 5% duty cycle, 1.4-Watt power and 0.15-s duration, and the spots were applied over the area of RPE leakage and all areas of pigmentary abnormality, including the fovea, if indicated [16]. Titration was performed to identify the minimal power abler to avoid any visible whitening reaction on the retina.

In the study of Arsan, STLT was performed by means of a 577-nm laser and using a 160-μm spot size diameter, 20-ms duration and 5% duty cycle, and also, in this case, the power was modulated so that, on ophthalmoscopy, no visible or detectable retinal changes were induced [17]. Fluorescein angiography (FA) was used to identify the area to be treated with confluent spots on the active leakage area and surrounding neighborhood one-spot-size area.

In the study with a long-term follow-up of more than three years, Kim et al. employed a 577-nm laser in multiple session and with the following settings: 100-μm spot diameter, 20-ms duration, 15% duty cycle and low power ranging from 200 to 400 mW [18]. In particular, the power was incrementally increased by 100 mW, depending on the resolution of the NSD at the monthly follow-up visits. The distribution of the contiguous laser spots covered the area of RPE leakage and all areas of NSD, including also the fovea.

Isik et al. applied STLT by means of a 577-nm and with the following settings: 160-μm spot diameter, a 200-ms duration and 5% duty cycle energy [19]. The power titration was also applied. The area of leakage was identified by means of FA; however, all areas of retinal edema on the optical coherence tomography (OCT) thickness map was also treated. The treatment grid included an area of 1000 micron centered on the fovea, with additional treatment on the leaking areas outside the central area.

**Figure 4:**
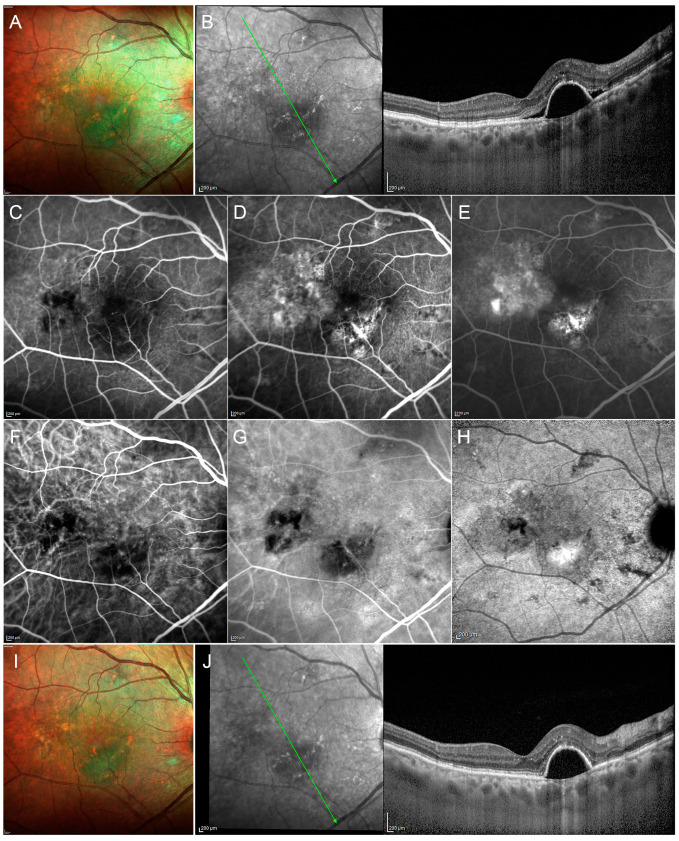
Multimodal imaging in a case of chronic CSC. Multicolor image (**A**) discloses multiple alterations together with the presence of fluid. Structural OCT scan through the fovea (green arrow) (**B**) shows a big pigment epithelium detachment (PED) with perilesional subretinal exudation and reflectivity changes localized at the level of the outer retinal bands. Fluorescein angiography discloses several hypofluorescent alterations in the early phase (**C**), becoming hyperfluorescent in the intermediate and late phases of the exam (**D**,**E**, respectively). Indocyanine green angiography highlights the changes of the choroidal vascular network in the early, intermediate and late phases (**F**–**H**, respectively). After photodynamic therapy with verteporfin, multimodal imaging discloses the regression of the subretinal fluid, with the permanence of the PED turning out to be stable in size (**I**,**J**, respectively).

At first impression, the STLT seems to offer great flexibility in the treatment procedure, allowing to operate in the context of this difficult retinal disorder with relative safety, even in the central treatment areas involving the fovea. In addition, the absence of any type of complication related to the treatment—specifically RPE atrophy and CNV occurrence—also in the study with the long-term follow-up makes it an attractive therapeutic approach, especially considering the chronic course of CSC with potential recurrences (Figure 4).

Large prospective studies with long-term follow-up are needed to confirm these preliminary results to validate the most effective treatment procedure, including the laser treatment settings but, also, the indications for the treatment criteria, referring, in particular, to the area to be treated—for example, the area of leakage on FA, the NSD area, the area of RPE decompensation or their combination. The new investigations should be integrated with an analysis of retinal sensitivity, which was lacking in all published studies with longer follow-ups.

### 2.3. Subthreshold Laser Treatment versus PDT

There are only a handful of comparative studies comparing the outcomes of STLT in comparison to photodynamic therapy with verteporfin (PDT) in prospective trials or retrospective studies (Table 2). Shortly, in PDT, a primarily nontoxic photosensibilizator—the verteporfin—is administered intravenously, its subsequent activation by means of low-energy, nonthermal laser leads to the formation of reactive oxygen species able to induce oxidative stress and cell death with secondary vascular damage. PDT has been hypothesized to cause choriocapillary hypoperfusion, possibly as a result of direct action on the choriocapillary endothelium and consequent choriocapillary occlusion, and leading to a reduction in choroidal congestion, vascular hyperpermeability and extravascular leakage.

Four retrospective studies analyzed the effects of STLT with a 577-nm wavelength or navigated/microsecond laser versus half-fluence PDT or half-dose PDT [21,22,23,24]. The follow-up ranged between six weeks and 17.4 months. BCVA outcomes were generally favorable in both treatment arms, with a registered stabilization or improvement between 0.03 and 0.2 LogMar and the study of Ozmert reporting a BCVA improvement of four ETDRS (Early Treatment Diabetic Retinopathy Study) letters in both groups. The results appeared also positive with regard to the complete resolution of NSD. Scholz et al. reported the lower proportion of NSD resolution, 21% in the half-dose PDT group and 36% in the STLT group; however, the follow-up was no longer than six weeks, probably a time interval too short to demonstrate the effects of both treatments. The proportion of NSD was significantly higher in the studies with longer follow-ups, with values ranging from 59% to 92% over a 6–17.4-month follow-up and broadly similar to resolution rates in the PDT groups. Retinal sensitivity was never investigated in all these studies.

Data on NSD recurrences are very difficult to evaluate for the different follow-up times and because most of the studies do not report precise values. As a consequence—also the second treatment administered during the study’s course of reduced PDT or STLT—it is not clearly correlated to the persistence of the NSD or the recurrence.

Of note, substantial differences exist in the protocols of the delivery of the STLT with regards to the treatment areas. In detail, the protocols were differently settled to treat the areas of leakage recognized as hot spots in fluorescein angiography (FA), or the areas of hyperfluorescence on indocyanine green angiography (ICGA) and the corresponding areas of leakage on FA or the edematous areas identified on OCT. Laser setting parameters were instead quite similar, with duty cycle 5%, spot diameter included between 100–200 microns, duration 200 ms and by delivering confluent spots and selecting the power after titration in a healthy area outside the area to be treated.

Finally, these retrospective studies seem converged to a common positive demonstration for the effectiveness of the STLT, also highlighting the numerous advantages over PDT, including the easy administration, the laser treatment management and the lower costs.

The efficacy of STLT and half-dose PDT for chronic CSC (cCSC) has been also evaluated in three prospective randomized clinical trials with short-term follow-ups.

Data from 33 patients with cCSC were reported in a six-month follow-up single-center study carried out by Ho et al. [25]. Eighteen and 15 subjects were allocated to STLT and to Half Dose-PDT groups, respectively. Both treatment arms displayed a significant improvement in BCVA and central retinal thickness, with no difference between the two groups at the baseline and final examinations. Unexpectedly, the percent data of complete NSD resolution at six months, as well as the number of recurrences over the follow-up, were not reported.

Similarly, the study of Kretz et al. reported a BCVA improvement over a four-month follow-up study without reporting precise data with regards to the NSD resolution [26].

The PLACE trial (Half-Dose Photodynamic Therapy versus High-Density Subthreshold Micropulse Laser Treatment in Patients with Chronic Central Serous Chorioretinopathy) represents the largest multicenter, randomized, clinical trial that has compared the anatomical and functional efficacy of HD-PDT and STLT treatments in a short-term follow-up [20]. In each treatment arm, 80 patients were initially included, with 67 subjects in the HD-PDT group and 66 subjects in the STLT group completing the seven–eight-month course of the study.

At the final visit, both groups showed an improvement in BCVA and in the 25-item National Eye Institute Visual Function Questionnaire (NEI-VFQ25) score without registering the differences between the two groups. Of note, a higher percentage of complete NSD resolution was described in the HD-PDT group compared with the STLT group (67.2% versus 28.8%), and a more significant improvement in central retinal sensitivity was also measured in the HD-PDT group. Recurrences occurred in four patients (5%) receiving HD-PDT and in one patient (1.3%) in the STLT group. No complications related to treatment were registered in both groups.

The authors stated that a half-dose PDT was superior to STLT for treating cCSC and yielded to a significantly higher proportion of subjects with complete NSD resolution and functional improvement.

The results of the PLACE trial have seriously questioned the efficacy of subthreshold laser application. Indeed, the PLACE study apparently showed the superiority of PDT in terms of subretinal fluid resolution, even with no statistically significant difference regarding the visual acuity improvement.

After data presentation, the PLACE trial received serious criticism, especially for the laser delivery methodology. In particular, STLT was not appropriately administered in the PLACE trial, making it a kind of “homeopathic” therapy rather than a real laser application [27,28]. The International Retinal Laser Society has recently offered simple guidelines to optimize the subthreshold laser application [29], more clearly specifying that a panmacular treatment (covering the whole macula between the vascular arcades) is mandatory, “painting” the whole macula with confluent laser spots several times over in a single treatment session to avoid any undertreatment. A clinical trial is currently ongoing (ClinicalTrials.gov Identifier: NCT04410861) to assess the effects of panmacular subthreshold laser for CSC.

### 2.4. STLT versus Conventional Laser Treatment

The efficacy and safety of STLT was compared with a threshold conventional laser (TCL) in central serous chorioretinopathy in a RCT with 12-weeks follow-up [30]. Inclusion criteria comprised a NSD lasting less than six months, and the leakage points detected on fluorescein angiography had to be located in ETDRS ring 2 or 3.

The Supra Scan 577 nm (Quantel Medical Sub-Liminal laser) was settled in micropulse or continuous laser mode by delivering 150–200 spots in the STLT group and 18–27 spots in the TCL group. In each group, 44 patients were included and completed the study. A BCVA improvement of six ETDRS letters was registered in both groups. Of note, TCL acted faster on the resorption of subretinal fluid, although a final no significant increased proportion of subjects with a complete resolution of NSD was identified in the TCL group (81% versus 64%). With regard to the safety profile, CNV occurred in one patient in the TCL group, whereas no RPE atrophy, pigmentation or migration were identified in the study population. A greater proportion of mild RPE depigmentation was detected in 33% of subjects in the TCL group compared with 12% patients in the STLT group.

Maruko et al. investigated the effects of the conventional laser (577-nm yellow laser, NIDEK MC-500) versus the STLT (577-nm micropulse yellow laser, IRIDEX IQ577) in a retrospective study performed on 28 patients [31]. The follow-up was very short, with a mean time of 3.4 months in the TCL group and 2.2 months in the STLT group. BCVA was almost normal at the baseline, and no visual improvement could be measured at the final visit. Complete resolution of NSD was observed in 66% and in 64% of cases in the TCL and in the STLT group, respectively. The final RPE modifications were evaluated by fundus autofluorescence, which allowed to detect RPE damage in the site of laser delivery in 10/10 eyes in the TCL group and one/nine in the SML-treated group.

A limitation of these studies includes the short-term follow-up unable to highlight the effects on NSD recurrences; in addition, the studies included only eyes with extrafoveal and typical leakage points in the TCL group and a small number of cases in the STLT group with leaking points under 500microns from the fovea.

### 2.5. STLT versus Eplerenone

**Figure 5:**
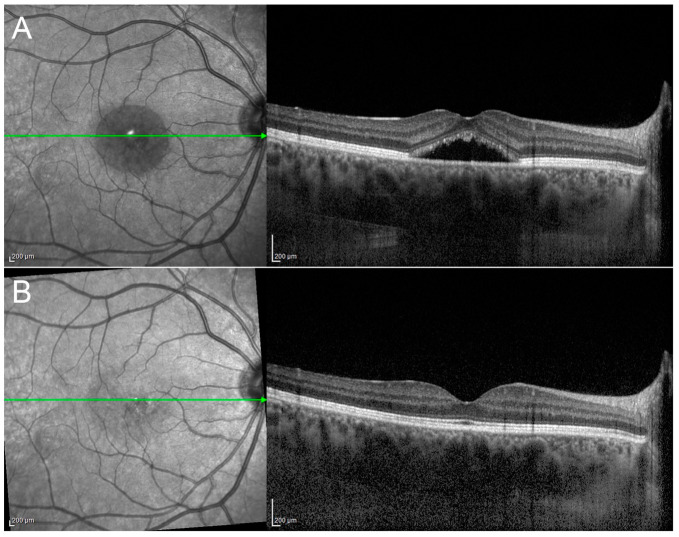
A case of acute CSC regression after eplerenone oral therapy. Structural OCT scan passing through the fovea (green arrow) shows a typical case of acute CSC, with a subfoveal fluid accumulation and an evident pachychoroid (**A**). After three months of eplerenone oral treatment, structural OCT discloses the complete regression of the subretinal fluid (**B**).

A potential role of an overaction of mineralocorticoid receptor pathways in choroidal vessels was suggested in the complex pathophysiology of cCSC, and many recent studies reported the efficacy of eplerenone and spironolactone in the treatment of cCSC (Figure 5).

Vignesh et al. reported the only retrospective comparative study evaluating the effects of eplerenone and STLT in a group of 48 eyes with cCSC [32]. Eplerenone was administered at a 25-mg daily dose for one month and then 50 mg daily for two months with monthly assessments of the serum potassium. STLT was performed by using a 577-nm micropulse yellow laser (IRIDEX IQ577) and delivering the treatment to the entire area of choroidal vascular hyperpermeability and including any focal/nonspecific leaks and area of RPE decompensation identified on FA and with the following settings: 5% duty cycle, 200-millisecond duration and 100-μm spot size.

After a median follow-up of eight months in the STLT group and four-and-a-half months in the eplerenone group, a complete NSD resolution was detected in 12/28 (42.8%) eyes in the STLT group and 4/20 (20%) in the eplerenone group. A final BCVA improvement of 0.14 LogMar was seen in the STLT group, whereas a slight worsening of 0.05 LogMar was observed in the eplerenone group. No data was reported about NSD recurrence.

It is difficult to share the authors’ conclusions on the efficacy of both methods in the treatment of cCSC. The difference in the median follow-up time does not allow to adequately assess the proportion of success in the resolution of NSD in the eplerenone group, and, probably, the initial difference in BCVA values between the two groups may have influenced the final results.

Unfortunately, no study investigated the effects of STLT and spironolactone in head-to-head comparative studies.

### 2.6. STLT versus Antivascular Endothelial Growth Factor (VEGF)

Although there is no documented evidence of a high vitreous titer of VEGF, many authors believe that anti-VEGF can still be used to regulate the choroidal hyperpermeability characterizing the cCSC. Koss et al. performed a comparative, controlled, prospective study conducted over a period of 10 months, comparing STLT versus bevacizumab [33]. A total of 52 eyes were randomly assigned to STLT (16 eyes) and intravitreal bevacizumab (10 eyes), with 26 eyes included in the observation group. The functional assessment demonstrated a mean BCVA improvement of six letters in the STLT group, a reduction of one and two letters, in the bevacizumab and control groups, respectively. Unfortunately, the authors did not report the proportion of eyes with complete NSD resolution, whereas the estimation of efficacy may be extrapolated by evaluating the residual leakage entity at the final examination. In detail, the leakage decreased significantly in the STLT group, with a residual proportion of eyes still showing activity at the final visit in 12.5%, compared with 60% in the bevacizumab group and 92% in the observational group.

### 2.7. Subvisible Damaging Laser

In addition to STLT, a second class of ophthalmoscopically subvisible/nonvisible laser treatments but minimally damaging exists, in which RPE cells are selectively destroyed through direct damage to melanosomes and obtained by mean of miscosecond/nanosecond applications of lasers. These lasers are currently called selective retinal laser treatments (SRT) [34,35,36,37,38,39,40].

Elsner et al. presented their preliminary experience on 27 patients with active CSC treated with SRT and using a pulsed double-Q-switched Nd-YLF prototype laser (λ = 527 nm) [40]. The settings of the laser were arranged as follows: pulse duration of 1.7 μs, laser spot size 200 μm, energies ranging from 100 to 350 μJ and, finally, each exposition was a train of 30 pulses. Since a selective retinal laser operates in the subvisible spectrum, the laser is integrated with a detector able to discriminate the optoacoustic signal product by expansion of the microbubbles inside the RPE cells and damaging the RPE cells itself. The optoacoustic signal is strongly correlated with the endpoint of the laser treatment and represents the “threshold” of the RPE damage. Such an anatomical finding and the validation of optoacoustic methodology are validated by means of fluorescein angiography, which is able to recognize the effects on RPE one hour after the laser delivery.

In this cohort of patients, a BCVA improvement was observed in all patients starting from two to three weeks after SRT. At a one-month examination, 85.2% of eyes showed a complete NSD resolution, and in the remaining eyes, the subretinal fluid was significantly reduced. Sixteen patients completing the three-months follow-up showed complete and persistent NSD resolution, no active leakage on FA and visual acuity 20/20 in all cases. Unfortunately, the authors did not report a categorization of outcomes according to focal or diffuse leakages or with reference to foveal positions.

Framme et al. provided additional data on the efficacy of SRT by distinguishing the acute CSC from the chronic CSC group [35]. In the first case, the SRT achieved similar results to those described by Elsner. In acute CSC, a single pinpoint leakage was treated, and a complete NSD resolution was obtained in 10/10 eyes in parallel to a BCVA improvement in all cases. Differently, most patients of the chronic form were characterized by diffuse leakage or a combination of diffuse leakage plus focal point, with only three patients with isolated pinpoints. At a three-month examination, NSD was resolved in only 19% (3/16) of cases; sic eyes received additional SRT sessions with higher energy levels, and five of them achieved a complete subretinal fluid reabsorption with a final 50% of success in all cohorts of patients with cCSC. It is very interesting that multiple sessions of SRT did not induce visible lesions to the retinal tissue.

The study of Kang further corroborates the beneficial effects of SRT in terms of visual acuity improvement and resolution of NSD, obtaining a significant 0.1-LogMar increase in BCVA and achieving a 100% (12/12 eyes) subretinal fluid resolution [34]. More interesting, the retinal sensitivity assessment on-site of the NSD and treated areas revealed a very small difference of only 3 dB between the site of laser application and the untreated adjacent region, confirming the absence of scotoma related to SRT, and with a parallel preserved photoreceptor integrity, as revealed by OCT in an all-SRT-treated site. Data on retinal sensitivity was also confirmed in the study of Yasui [36].

Additional studies provided similar results, providing evidence of the efficacy of SRT for acute and chronic forms of CSC. However, as is the case for all innovative therapies, few data are available in the literature to evaluate the long-term effects, leaving important gaps such as the effects of repeated treatments and the management of the recurrences of activity of this complex retinal disorder.

## 3. Discussion

The laser treatment of CSC gradually moved from conventional lasers with visible endpoint and retinal damage to subthreshold lasers, confined to the RPE in order to avoid any retinal harm. CSC subforms more pathogenetically related to alterations of the RPE may fare well with subthreshold laser treatment, allowing also repeated treatments without the risk of atrophic degeneration. Further studies are warranted to more clearly define the role of modern laser treatments in CSC.

## 4. Materials and Methods

The authors carried out an electronic search for relevant articles in PubMed from inception until August 2020. The workflow was arranged in compliance with the guidelines of the preferred reporting items for systematic reviews and meta-analyses (PRISMA) model. Main keywords used in combination included “central serous chorioretinopathy”, “laser treatment”, “subthreshold laser treatment”, “nondamaging laser treatment”, “selective laser” and “microsecond laser”. Moreover, secondary keywords such as “photodynamic therapy”, “antagonists of mineralocorticoid receptors”, “anti-VEGF drugs” and “transpupillary thermotherapy” were included in combination to find out the comparative studies.

Two authors (P.I. and A.A.) independently evaluated the preliminary results of the research using the title of the manuscript and the abstract as discriminating elements. A first exclusion was applied for review papers, case reports or small case series and articles not written in English. However, the pilot studies published in the first three years from the first articles published with the topic laser/CSC were considered. The resulting references were evaluated in order to identify the relevant studies, focusing especially on randomized clinical trials, prospective studies, comparative studies and retrospective studies with large samples.

The final list produced by the two authors was then collectively evaluated and discussed with the other authors, assessing the relevance of the studies with the aims of the current research; in particular, all the authors were aware that there was no claim to produce a meta-analysis but to provide a practical review of the literature currently available, with the aim of offering points of discussion and personal critical review.

The first selection identified 68 manuscripts from which 20 papers were excluded: case report, review and nonEnglish. Finally, for the purposes of the current research, 30 manuscripts were included, divided within the following categorized topics: conventional laser treatment (9 manuscripts), subthreshold laser treatment (6 manuscripts), subthreshold laser treatment versus photodynamic therapy with verteporfin (PDT) (6 manuscripts), subthreshold laser treatment versus mineralocorticoids antagonist (1 manuscript), subthreshold laser treatment versus anti-VEGF (1 manuscript) and selective laser treatment (7 manuscript).

## Figures and Tables

**Table 1:** Subthreshold laser treatment for central serous chorioretinopathy. Summary of studies with at least 12-months follow-up.

Authors (P/R)	F-UP	N° Patients	BCVA	RS (dB)	NSD Resolution	NSD Recurrences	N° Treatments	Side Effects
Kim [18] (R)	3.7 years	27	+0.18 LogMar	-	81.5%	6 (22%)	2.5	None
Isik [19] (R)	11.4 months	58	+0.22 LogMar	-	67.2%	6 (10.3%)	1–2	None
Arsan [17] (P)	17 months	39	+0.40 LogMar	-	92.3%	9 (23%)	1.4 (1–4)	None
Lutrull [16] (R)	14 months	11	Improved in all pts	-	100%	1	1	None
Lanzetta [11] (P)	14 months	24	+0.10 LogMar	-	75%	-	1	None

P/R: prospective/retrospective. BCVA: best-corrected visual acuity. RS: retinal sensitivity. NSD: neurosensory detachment. F-UP: follow-up. N°: number of. dB: decibel.

**Table 2:** Summary of comparative studies with adapted photodynamic therapy (PDT) protocols versus subthreshold laser treatments (STLTs) for chronic central serous chorioretinopathy.

Authors (P/R)	Protocol	F-UP	N° pts	BCVA	NSD Resolution	NSD Recurrences	N° Treatment	Side Effects
PLACE trial [20] (P)	HD-PDT	7–8 months	67	+6.7 letters	67.2 %	4 (5%)	1–2	0
Scholz [21] (R)	HD-PDT	6 weeks	58	+0.04 Logmar	21%	-	1–2	1 CNV
	STLT-577 nm	6 weeks	42	+0.08 Logmar	36%	-	1–2	0
Ozmert [22] (R)	HF-PDT	1 year	18	+4 letters	72%	1	1–2	0
	STLT-Microsecond	1 year	15	+4 letters	80%	2	1	1 Hypofluorescent spot
Roca [23] (R)	HD-PDT	17.4 months	67	+0.03 Logmar	95.5%	-	1 2 (5 eyes) 3 (1 eye)	1 CNV
	STLT-577 nm	15.8 months	92	+0.21 Logmar	92.4 %	-	1 2 (12 eyes) 3 (2 eyes) 4 (2 eyes)	0
Ntomoka [24] (R)	HF-PDT	6 months	22	+0.1 Logmar	21.7%	-	1	0
	STLT-577 nm	6 months	23	+0.2 Logmar	59%	-	1	0
Ho [25] (P)	HD-PDT	6 months	15	+0.14 Logmar	-	-	1	-
	STLT-577 nm	6 months	18	+0.20 Logmar	-	-	1	-
	STLT-810nm	7–8 months	66	+4.4 letters	28.8%	1 (1.3)	1–2	0

P/R: prospective/retrospective. BCVA: best-corrected visual acuity. NSD: neurosensory detachment. CNV: choroidal neovascularization. PDT: Photodynamic therapy. HD: Half-dose. HF: Half-fluence. STLT: Subthreshold laser treatment. PLACE trial (Half-Dose Photodynamic Therapy versus High-Density Subthreshold Micropulse Laser Treatment in Patients with Chronic Central Serous Chorioretinopathy).

## References

[1] Iacono P., Battaglia Parodi M., Falcomatà B., Bandello F. (2015). Central Serous Chorioretinopathy Treatments: A Mini Review. Ophthalmic Res..

[2] Inomata H. (1975). Wound healing after xenon arc photocoagulation in the rabbit retina. Identification of the proliferating cells in the lesion by light and electron microscopic autoradiography using 3H-thymidine. Ophthalmologica.

[3] Johnson R.N., McNaught E.I., Foulds W.S. (1977). Effect of photocoagulation on the barrier function of the pigment epithelium. II. A study by electron microscopy. Trans. Ophthalmol. Soc. UK.

[4] Daruich A., Matet A., Dirani A., Bousquet E., Zhao M., Farman N., Jaisser F., Behar-Cohen F. (2015). Central serous chorioretinopathy: Recent findings and new physiopathology hypothesis. Prog. Retin. Eye Res..

[5] Lim J.W., Kang S.W., Kim Y.-T., Chung S.E., Lee S.W. (2011). Comparative study of patients with central serous chorioretinopathy undergoing focal laser photocoagulation or photodynamic therapy. Br. J. Ophthalmol..

[6] Leaver P., Williams C. (1979). Argon laser photocoagulation in the treatment of central serous retinopathy. Br. J. Ophthalmol..

[7] Brancato R., Pratesi R., Leoni G., Trabucchi G., Vanni U. (1989). Histopathology of diode and argon laser lesions in rabbit retina: A comparative study. Investig. Ophthalmol. Vis. Sci..

[8] Loo R.H., Scott I.U., Flynn H.W., Gass J.D., Murray T.G., Lewis M.L., Rosenfeld P.J., Smiddy W.E. (2002). Factors associated with reduced visual acuity during long-term follow-up of patients with idiopathic central serous chorioretinopathy. Retina.

[9] Luttrull J.K., Dorin G. (2012). Subthreshold Diode Micropulse Laser Photocoagulation (SDM) as Invisible Retinal Phototherapy for Diabetic Macular Edema: A Review. Curr. Diabetes Rev..

[10] Verma L., Sinha R., Venkatesh P., Tewari H.K. (2004). Comparative evaluation of diode laser versus argon laser photocoagulation in patients with central serous retinopathy: A pilot, randomized controlled trial [ISRCTN84128484]. BMC Ophthalmol..

[11] Lanzetta P., Furlan F., Morgante L., Veritti D., Bandello F. (2008). Nonvisible subthreshold micropulse diode laser (810 nm) treatment of central serous chorioretinopathy: A pilot study. Eur. J. Ophthalmol..

[12] Chen S.N., Hwang J.F., Tseng L.F., Lin C.J. (2008). Subthreshold diode micropulse photocoagulation for the treatment of chronic central serous chorioretinopathy with juxtafoveal leakage. Ophthalmology.

[13] Scholz P., Ersoy L., Boon C.J., Fauser S. (2015). Subthreshold micropulse laser (577 nm) treatment in chronic central serous chorioretinopathy. Ophthalmologica.

[14] Yadav N.K., Jayadev C., Mohan A., Vijayan P., Battu R., Dabir S., Shetty B., Shetty R. (2015). Subthreshold micropulse yellow laser (577 nm) in chronic central serous chorioretinopathy: Safety profile and treatment outcome. Eye.

[15] Kim J.Y., Park H.S., Kim S.Y. (2015). Short-term efficacy of subthreshold micropulse yellow laser (577 nm) photocoagulation for chronic central serous chorioretinopathy. Graefe’s Arch. Clin. Exp. Ophthalmol..

[16] Luttrull J.K. (2016). Low-intensity/high-density subthreshold diode micropulse laser for central serous chorioretinopathy. Retina.

[17] Arsan A., Kanar H.S., Sonmez A. (2018). Visual outcomes and anatomic changes after sub-threshold micropulse yellow laser (577 nm) treatment for chronic central serous chorioretinopathy: Long-term follow-up. Eye.

[18] Kim Y.J., Kim S.Y., Ha S., Moon D., Seong S., Kwon O.W., Park H.S. (2019). Short-duration multiple-session subthreshold micropulse yellow laser (577 nm) for chronic central serous chorioretinopathy: Results at 3 years. Eye.

[19] Işık M.U., Değirmenci M.F.K., Sağlık A. (2020). Efficacy of the subthreshold micropulse yellow wavelength laser photostimulation in the treatment of chronic central serous chorioretinopathy. Int. J. Ophthalmol..

[20] Van Dijk E.H., Fauser S., Breukink M.B., Blanco-Garavito R., Groenewoud J.M., Keunen J.E., Peters P.J., Dijkman G., Souied E.H., MacLaren R.E. (2018). Half-dose photodynamic therapy versus high-density subthreshold micropulse laser treatment in patients with chronic central serous chorioretinopathy: The PLACE trial. Ophthalmology.

[21] Scholz P., Altay L., Fauser S. (2016). Comparison of subthreshold micropulse laser (577 nm) treatment and half-dose photodynamic therapy in patients with chronic central serous chorioretinopathy. Eye.

[22] Özmert E., Demirel S., Yanık Ö., Batıoğlu F. (2016). Low-Fluence Photodynamic Therapy versus Subthreshold Micropulse Yellow Wavelength Laser in the Treatment of Chronic Central Serous Chorioretinopathy. J. Ophthalmol..

[23] Roca J.A., Wu L., Fromow-Guerra J., Rodríguez F.J., Berrocal M.H., Rojas S., Lima L.H., Gallego-Pinazo R., Chhablani J., Arevalo J.F. (2018). Yellow (577 nm) micropulse laser versus half-dose verteporfin photodynamic therapy in eyes with chronic central serous chorioretinopathy: Results of the Pan-American Collaborative Retina Study (PACORES) Group. Br. J. Ophthalmol..

[24] Ntomoka C.G., Rajesh B., Muriithi G.M., Goud A., Chhablani J. (2018). Comparison of photodynamic therapy and navigated microsecond laser for chronic central serous chorioretinopathy. Eye.

[25] Ho M., Lai F.H.P., Ng D.S.C., Iu L.P.L., Chen L.J., Mak A.C.Y., Yip Y., Cheung C., Young A.L., Brelen M. (2020). Analysis of choriocapillaris perfusion and choroidal layer changes in patients with chronic central serous chorioretinopathy randomised to micropulse laser or photodynamic therapy. Br. J. Ophthalmol..

[26] Kretz F.T., Beger I., Koch F., Nowomiejska K., Auffarth G.U., Koss M.J. (2015). Randomized Clinical Trial to Compare Micropulse Photocoagulation Versus Half-dose Verteporfin Photodynamic Therapy in the Treatment of Central Serous Chorioretinopathy. Ophthalmic Surg. Lasers Imaging Retin..

[27] Battaglia Parodi M., Iacono P. (2019). Re: Van Dijk et al.: Half-dose photodynamic therapy versus high-density subthreshold micropulse laser treatment in patients with chronic central serous chorioretinopathy: The PLACE trial (*Ophthalmology*
**2018**, *125*, 1547–1555). Ophthalmology.

[28] Luttrull J.K. (2020). Comment on: Focal and Diffuse Chronic Central Serous Chorioretinopathy Treated with Half-Dose Photodynamic Therapy or Subthreshold Micropulse Laser: PLACE Trial Report No. 3. Am. J. Ophthalmol..

[29] Keunen J.E.E., Battaglia Parodi M., Vujosevic S., Luttrull J.K. (2020). International Retinal Laser Society Guidelines for Subthreshold Laser Treatment. Transl. Vis. Sci. Technol..

[30] Sun Z., Huang Y., Nie C., Wang Z., Pei J., Lin B., Zhou R., Zhang J., Chong V., Liu X. (2020). Efficacy and safety of subthreshold micropulse laser compared with threshold conventional laser in central serous chorioretinopathy. Eye.

[31] Maruko I., Koizumi H., Hasegawa T., Arakawa H., Iida T. (2017). Subthreshold 577 nm micropulse laser treatment for central serous chorioretinopathy. PLoS ONE.

[32] Vignesh T.P., Maitray A., Sen S., Chakrabarti A., Kannan N.B., Ramasamy K. (2020). Subthreshold Micro-Pulse Yellow Laser and Eplerenone Drug Therapy in Chronic Central Serous Chorio-Retinopathy Patients: A Comparative Study. Semin. Ophthalmol..

[33] Koss M.J., Beger I., Koch F.H. (2012). Subthreshold diode laser micropulse photocoagulation versus intravitreal injections of bevacizumab in the treatment of central serous chorioretinopathy. Eye.

[34] Kang S., Park Y.G., Kim J.R., Seifert E., Theisen-Kunde D., Brinkmann R., Roh Y.J. (2016). Selective Retina Therapy in Patients with Chronic Central Serous Chorioretinopathy: A Pilot Study. Medicine (Baltim).

[35] Framme C., Walter A., Berger L., Prahs P., Alt C., Theisen-Kunde D., Kowal J., Brinkmann R. (2015). Selective Retina Therapy in Acute and Chronic-Recurrent Central Serous Chorioretinopathy. Ophthalmologica.

[36] Yasui A., Yamamoto M., Hirayama K., Shiraki K., Theisen-Kunde D., Brinkmann R., Miura Y., Kohno T. (2017). Retinal sensitivity after selective retina therapy (SRT) on patients with central serous chorioretinopathy. Graefe’s Arch. Clin. Exp. Ophthalmol..

[37] Kim Y.J., Lee Y.G., Lee D.W., Kim J.H. (2018). Selective Retina Therapy with Real-Time Feedback-Controlled Dosimetry for Treating Acute Idiopathic Central Serous Chorioretinopathy in Korean Patients. J. Ophthalmol..

[38] Park Y.G., Kang S., Kim M., Yoo N., Roh Y.J. (2017). Selective retina therapy with automatic real-time feedback-controlled dosimetry for chronic central serous chorioretinopathy in Korean patients. Graefe’s Arch. Clin. Exp. Ophthalmol..

[39] Klatt C., Saeger M., Oppermann T., Pörksen E., Treumer F., Hillenkamp J., Fritzer E., Brinkmann R., Birngruber R., Roider J. (2011). Selective retina therapy for acute central serous chorioretinopathy. Br. J. Ophthalmol..

[40] Elsner H., Pörksen E., Klatt C., Bunse A., Theisen-Kunde D., Brinkmann R., Birngruber R., Laqua H., Roider J. (2006). Selective retina therapy in patients with central serous chorioretinopathy. Graefe’s Arch. Clin. Exp. Ophthalmol..

